# Information on medicines: Does independence from industry influence matter?

**DOI:** 10.4102/phcfm.v16i1.4522

**Published:** 2024-03-29

**Authors:** Barbara Mintzes

**Affiliations:** 1 School of Pharmacy and Charles Perkins Centre, Faculty of Medicine and Health, The University of Sydney, Sydney, Australia

This editorial in the *African Journal of Primary Health Care & Family Medicine* introduces a new series of bulletins on drug treatments, the *Therapeutics Letter*.^1^ The Therapeutics Initiative, a team based at the University of British Columbia, in Vancouver, Canada, produces the *Therapeutics Letter*, with the aim to present brief, critical summaries of the evidence on outcomes of drug treatments to help guide clinical practice. The focus of this series is on conditions and treatments of relevance to primary care in Africa. In due course, we also plan to develop our own evidence synthesis in collaboration with local evidence-based healthcare experts and clinical pharmacology colleagues. The new series will be curated and edited by Prof. Michael Pather.

The Therapeutics Initiative is a long-time member of the International Society of Drug Bulletins (ISDB), a global network dedicated to producing critical, evidence-based analyses of drug treatments that are independent of industry influence. All ISDB members have strong conflict of interest policies: they accept no funding from pharmaceutical or device companies. Neither editorial teams nor authors of articles that could influence clinical practice have any industry funding.

Why should medicines information be independent of industry? After all, drug companies develop medicines, carry out trials to get them to market, and keep tabs on post-market safety. Aren’t they the experts on their own products?

Drug companies do have extensive information on their medicines. They also contribute to public health, by developing, manufacturing and distributing medicines that manage chronic conditions, ease symptoms, improve quality of life and are sometimes lifesaving. However, these companies have a primary fiduciary responsibility to shareholders to maintain profitability. This creates a consistent bias towards presenting their products in a positive light. It is also easier to develop ‘me-too’ drugs than truly innovative new products. And sometimes the mechanism of action of a new medicine is innovative, but its effects on health are mediocre at best. To expand sales, marketing often trumps science. This makes sense commercially, but not for patient health.

**FIGURE 1:**
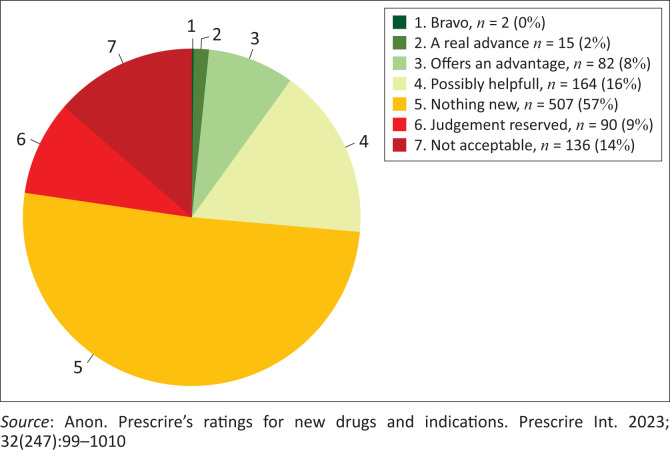
Prescrire International ratings for new medicines, 2013–2022 (*n* = 996).

One ISDB member, *La Revue Prescrire* (English edition *Prescrire International*), evaluates every new medicine marketed in France. Figure 1 reports their assessments over 10 years on a 7-point scale from ‘bravo’ to ‘not acceptable’.^2^ Around 10% had substantial advantages; another 16% had modest additional benefit; 51% were me-too’s, no better and no worse; and 14% had worse safety and/or effectiveness.

*Prescrire* also publishes an annual list of ‘Drugs to Avoid’ to guide better care. Analyses in Canada,^3^ Australia^4^ and the United Kingdom^5^ have highlighted how often these ‘Drugs to Avoid’ are prescribed. Denosumab (Prolia), for example, is often prescribed first-line for osteoporosis, but is no more effective than other osteoporosis drugs in preventing fractures and has a poor safety profile.^6^

For access to medicines, the gap between commercial pressures and health needs is widely recognised, with many lifesaving medicines not reaching those most in need, as occurred in the 1990s with human immunodeficiency virus (HIV) and/or acquired immune deficiency syndrome (AIDS) medicines, and recently with high-priced cancer medicines.

Less is known about commercial limits to access to lifesaving information. Internal documents released in legal cases indicate that Merck, manufacturer of the arthritis drug rofecoxib (Vioxx), had clinical trial evidence in 2001 of increased risks of death, but this information only became public years later.^7^ In the interim, many patients had heart attacks or died unnecessarily. A Spanish ISDB bulletin highlighted rofecoxib’s cardiovascular risks, referring to manipulation of the science, and was sued unsuccessfully by Merck.^8^ Similarly, although much has been done to improve clinical trial transparency, too often negative results remain hidden.^9^ Industry-sponsored continuing medical education has also been credited with fuelling the epidemic of opioid-related deaths in North America through inaccurate messages that newer opioids are nonaddictive, chronic pain is undertreated, and it is a ‘chronic disease’ for which long-term opioids use is appropriate.^10^

Commercial influence can also exaggerate drug benefits. A Cochrane systematic review found that industry-funded studies were 27% more likely (risk ratio [RR] 1.27; 95% confidence interval [CI] 1.17–1.37) to have efficacy results favourable to the tested products than non-industry-funded studies.^11^

Pharmaceutical industry influence has been called ‘the elephant in the room’ in medicine, omnipresent but never discussed, with around 60% of medical research globally funded by industry,^12^ most patient groups industry-funded,^13^ many doctors receiving industry payments^14^ and a strong influence on policy.^15^

What is the solution? One important step is to provide clinicians and patients with access to independent evidence-based information on the benefits and harms of drug treatments and their place in therapy. As a global network of independent bulletins, ISDB supports this goal through high editorial standards for accurate, up-to-date medicines information, by facilitating communication between members, training and information exchange, and through policy advocacy (https://www.isdbweb.org/).

International Society of Drug Bulletins currently has members in Europe, Asia, and North and South America but no members in Africa. We welcome new members and are thrilled about this initiative by the *African Journal of Primary Health Care & Family Medicine* to republish selected *Therapeutics Letters*. I hope you enjoy this series and find it useful to your practice.
